# Transport of Pigs of Two Market Weights at Two Space Allowances: Effects on Behaviour, Blood Parameters, and Meat Quality under Summer and Winter Conditions

**DOI:** 10.3390/ani13172767

**Published:** 2023-08-30

**Authors:** Jessica Gonçalves Vero, Nicolas Devillers, Ana Maria Bridi, Kyle A. T. Moak, Gizella Aboagye, Guilherme Agostinis Ferreira, Jansller Luiz Genova, Sabine Conte, Luigi Faucitano

**Affiliations:** 1 Agriculture and Agri-Food Canada, Sherbrooke Research and Development Centre, 2000 College Street, Sherbrooke, QC J1M 0C8, Canada; jgveroo@gmail.com (J.G.V.); nicolas.devillers@agr.gc.ca (N.D.); sabine.conte@agr.gc.ca (S.C.); 2 Departamento de Zootecnia, Universidade Estadual de Londrina, Londrina 86051-990, Brazil; ambridi@uel.br (A.M.B.); guilherme.agostinis@uel.br (G.A.F.); 3 Department of Animal Science, University of Saskatchewan, Saskatoon, SK S7N 5B4, Canada; kyle.moak@usask.ca; 4 European Food Safety Authority (EFSA), Via Carlo Magno 1A, 43126 Parma, Italy; gizi.aboagye@gmail.com; 5 Departamento de Zootecnia, Universidade Federal de Viçosa, Viçosa 36570-900, Brazil; jansllerg@gmail.com

**Keywords:** behaviour, meat quality, pigs, slaughter weight, space allowance, stress, summer, transport, winter

## Abstract

**Simple Summary:**

Increasing the slaughter weight of pigs can create challenges in handling and transport, affecting the welfare and the final meat quality. Heavier pigs have different space requirements to fit their physical and thermal needs. This research was conducted through two separate experiments with the aim of investigating the effects of two slaughter weights (120 and 140 kg) and two space allowances during summer and winter transports, i.e., 0.54 and 0.62 m^2^/pig and 0.44 m^2^/pig or 0.50 m^2^/pig, respectively, on the behavioural and physiological response, and carcass and meat quality of pigs. Overall, the effects of providing different space allowances during summer transports on within-truck ambient conditions, post-transport pigs’ welfare, and carcass and meat quality are similar, regardless of the slaughter weight. In summer loads, increased slaughter weight is associated with more aggressive pigs under mixed group conditions in lairage and a higher risk of DFD-like pork meat resulting from fatigue at slaughter. Winter transport results may have been biased by the short journey and the truck condition not travelling at its full load capacity. Further research aiming for longer journeys (>3 h) is needed to validate the space allowances applied in this study for heavier pigs during summer and winter conditions.

**Abstract:**

This study was carried out through two separate experiments aiming at evaluating the effects of two space allowances (0.54 and 0.62 m^2^/pig in summer and 0.44 m^2^/pig and 0.50 m^2^/pig in winter) on the behavioural and physiological response, and meat quality of pigs of two slaughter weights (120 kg and 140 kg). In summer, higher blood haematocrit levels were found at slaughter in heavier pigs transported at a smaller space allowance (*p* = 0.04). During lairage, pigs transported at a smaller space allowance started fighting later (*p* = 0.04). Fighting behaviour was greater in heavier pigs (*p* ≤ 0.05), whilst their drinking activity was lower (*p* < 0.05). This resulted in greater exsanguination blood CK levels (*p* < 0.01) and drier hams (*p* = 0.05) in heavier pigs. In winter, only lower space allowance influenced some meat quality traits (*p* < 0.05), but these effects were minor. The effects of space allowance during summer transports on within-truck ambient conditions, post-transport pigs’ welfare, and meat quality are similar. Mixing heavier pigs may result in greater aggressiveness and more fatigue-related meat quality variation during summer. Overall, winter transport results may have been biased by the short journey and within-truck load distribution.

## 1. Introduction

Global pig meat production is expected to grow 13% over the coming decade, due to growth in developing countries, accounting for 33% of the growth in total global meat production [1]. The worldwide increase in slaughter weight of modern pigs may contribute to meeting this greater demand for pork meat. The profitability of heavier pig production for the pork chain economy is in terms of dilution of fixed production costs through the reduction of the total number of pigs required to produce a given quantity of pork [2], reduced costs for animal health control due to lower need for vaccine protection resulting from a more developed immune system [3], improvement of carcass yields and meat to bone ratio [4], and greater profits when pig prices are high or when finishing feed prices are relatively low [5]. For all these reasons, the average slaughter weight in Canada has increased from 105 kg in the late 1980s to the present 130 kg, with a major increase (+17 kg) between 2005 and 2010 [6]. It has been forecasted that market weights well above 130 kg will be attained over the next few years based on the average weight increase of 0.6 kg/year [7].

However, the increased weight and size of these pigs also create challenges to handling and transportation systems. Transport represents a stressful stimulus for the animal. It is well known that, under stressful stimuli, the body must find a new dynamic equilibrium, and this requires several adaptive body responses. Transport can result in a disruption of homeostasis, with a direct effect on animal health status and the physical performance of the animals [8,9]. The degree of stress during transport appears greater in heavier pigs compared with lighter pigs based on their decreased ease of handling during loading [10], higher salivary cortisol concentrations, and heart rate during loading and transport [11], which result in an increased risk of DOA (dead on arrival) during transport and bruised carcasses [12].

Based on the monitoring of more than 12,000 trailer loads, Fitzgerald et al. [13] concluded that floor space allowance alone contributed more to the variation of in-transport losses than other transport factors, such as the driver, handling staff, ambient conditions, and loading and wait time before unloading. In Canada, a minimum space allowance of 0.44 m^2^/130 kg is the legal requirement [14] to ensure that pigs can stand and lie down to rest in their natural position without being disturbed and pushed to continually change their position during transport. However, it has been demonstrated that pigs of modern genetics, being larger and heavier, may need a greater minimal space (approx. 0.49 m^2^/110 kg) to entirely lie down in a full lateral recumbent position on the truck floor [15]. This new information implies that transporting pigs at the presently recommended space allowance may result in overcrowding and a greater risk of bruises, injuries, fatigue, and, eventually, DOA.

Due to their increased size, heat output, poor dissipation rate, large muscle mass, and lower cardiac output [16,17,18], heavier pigs have different space requirements to meet their physical and thermal needs. As a result of their greater size, the Canadian recommendation for minimum space allowances, e.g., 0.50 m^2^/pig [14], for the transport of heavier pigs (140 kg), may result in overcrowding [19] and a greater risk of death during transport [20].

There is clear evidence that both warm and cold temperature conditions are challenging for pigs during transport as indicated by decreased ease of handling at loading [21,22] and increased physiological response, i.e., greater blood lactate, creatine kinase (CK) and cortisol concentrations, and heart rate [23,24,25], which may explain the greater proportion of pig losses [12], and pale and exudative (PSE) or dark, firm, and dry (DFD) pork meat [26,27] and increased incidence of carcass bruising [23,28] reported during summer and winter.

For these reasons, to meet their different physical and thermal needs, it is recommended to transport heavier pigs (from 114 kg to 182 kg) at increasingly greater minimum floor space from 0.46 to 0.65 m^2^/pig during summer and 0.40 m^2^/pig to 0.61 m^2^/pig during winter [29]. However, neither existing guidelines [29] nor codes of practice [14] are science-based.

This study unfolded through two separate transport trials whose objective was to evaluate the effects of two space allowances, i.e., 0.54 and 0.62 m^2^/pig and 0.44 m^2^/pig and 0.50 m^2^/pig, during summer and winter transport conditions, respectively, on the behavioural and physiological response, and carcass and meat quality of pigs weighing 120 and 140 kg on average.

## 2. Materials and Methods

All experimental procedures performed in these experiments were approved by the institutional animal care committee (Approval Number: 565) at the Agriculture and Agri-Food Canada (AAFC) Sherbrooke Research and Development Centre (Sherbrooke, QC, Canada) based on the current guidelines of the Canadian Council on Animal Care [30].

### 2.1. Animals and Treatments

#### Summer Transport Trials

**Figure 1:**
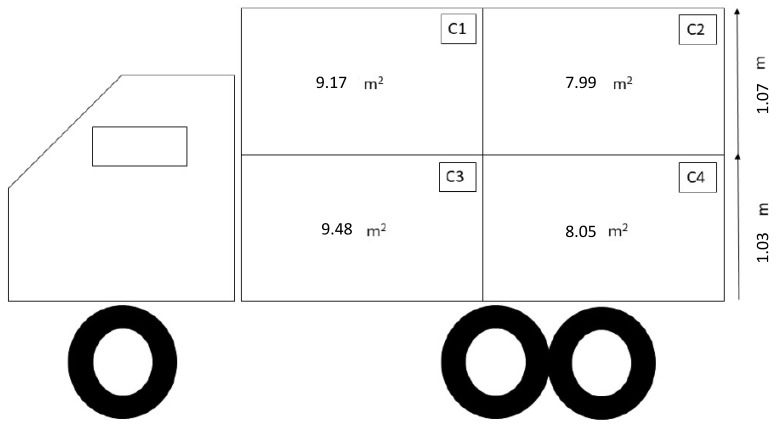
Representation of the truck design, including compartment dimensions, used for transport during summer.

A total of 359 immunologically castrated (Improvest^®^, Zoetis Canada, Kirkland, QC, Canada) boars and gilts of the same genetics (progeny of DNA 600 sires mated with Topigs TN70 dams) were shipped in six loads (five loads of 60 pigs and one load of 59 pigs) from a commercial finishing farm to a commercial abattoir (2 h travel) during the summer season (August to mid-September 2019; average temperature was 17.1 °C, ranging from 8.5 to 20.1 °C). Pigs were transported in the early morning (03:00 h AM) using a double-decked passively ventilated truck, featuring a fully hydraulic top deck and four compartments (two per deck; Figure 1).

The animals were distributed according to a 2 × 2 factorial arrangement, where the factors were the space allowance in the truck (0.54 and 0.62 m^2^/pig) and the body weight (120 ± 5 kg and 140 ± 5 kg). These space allowances were applied with respect to the Canadian Transport Codes of Practice [14], which recommends increasing the minimal space allowance by 25% during warm ambient conditions for transporting of pigs of these body weights. The final average body weight of pigs selected for the study was 123.7 and 142.3 kg for lighter and heavier weight groups, respectively.

Considering the previously reported effects of compartment location on internal truck ambient conditions and pigs’ response to handling and transport stress [12], space allowance × slaughter weight combinations were rotated across the four compartments for each shipment or replication (Table 1).

On the day prior to each shipment, 24 focal immunocastrated boars (six/truck compartment or space allowance × market weight combination), totalling 144 focal immunocastrated boars through the whole experiment, were selected from different finishing pens, weighed, and tagged in both ears for identification during blood collection at slaughter and carcass meat quality measurements, and were returned to the finishing pen where they were sorted out from during loading.

In preparation for transport, feed was withdrawn from all pigs for approximately 11 h before loading (approximately 15 h total fasting time at slaughter).

On the day of transportation, pigs, including focal ones, were loaded in small mixed groups of 5–10 pigs using paddles and sorting boards only. The truck left the farm upon completion of the loading process (wait-at-farm: 9 min on average, ranging from 4 to 14 min) to avoid the effect of the lack of passive ventilation on internal ambient conditions [31,32] and pig’s thermal comfort [33,34] during stationary phase. All selected pigs were fit for transport.

During transport, the side panels were kept fully open to allow passive ventilation. The truck was also bedded with fresh wood shavings. Due to unforeseen circumstances, the assigned driver was replaced for the last two shipments.

Pigs were unloaded almost immediately upon arrival at the abattoir (wait-at-abattoir: 5 min on average, ranging from 3 to 9 min) in groups of 13 pigs, which included 6 focal pigs, using paddles, and were driven to separate lairage pens based on the treatment combination or truck compartment. No DOA or non-ambulatory pigs were reported at unloading. No mixing of pigs between compartments/treatment combinations occurred. As the size of the truck compartments and lairage pens differed, to keep a constant stocking density (0.70 m^2^/pig) in the lairage pen, the truck compartment group size was reduced to 13 pigs/group (including the six focal pigs) in each lairage pen. Pigs were kept in lairage for 2.5 h, on average, with free access to water.

After lairage, pigs were driven using paddles along the alleys and an automatic push gate system in the last chute feeding the CO_2_ gas stunner (Frontmatec AS, M7-RR-S1, Kolding, Denmark). Immediately upon exiting the stunner, pigs were shackled and exsanguinated in the vertical position.

### 2.2. Winter Transport Trials

**Figure 2:**
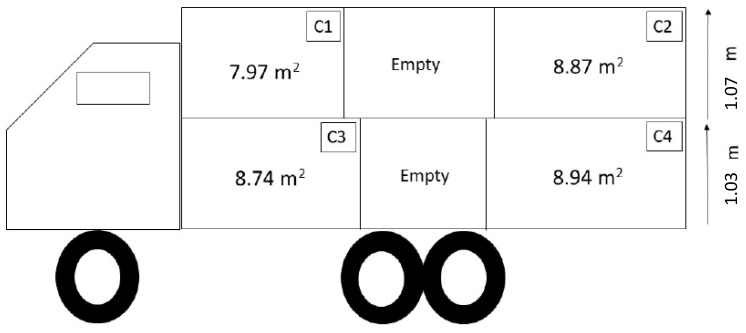
Representation of the truck design, including compartment dimensions, used for transport during winter.

A total of 295 immunologically castrated (Improvest^®^, Zoetis Canada, Kirkland, QC, Canada) boars and gilts of the same genetics (progeny of PIC L42 sires mated with PIC L8000 dams) were shipped in four loads (two loads of 75 pigs, one load of 73 pigs, and one load of 72 pigs) from a commercial finishing farm to a commercial abattoir (1 h travel) during the winter season (February to March 2020; average ambient air temperature 0.4 °C, ranging from −5.3 °C to 6.8 °C). Pigs were transported in the early morning (03:50 h AM) using a double-decked hydraulic passively ventilated truck, featuring six compartments, of which only four (two in the front and two in the rear of each deck) were used in the experiment. The two middle compartments were kept empty as the farm did not have enough volume to fill them with pigs (Figure 2).

The animals were distributed according to a 2 × 2 factorial arrangement, where the factors were the space allowance in the truck (0.44 m^2^/pig and 0.50 m^2^/pig) and the pig’s body weight (120 ± 5 kg and 140 ± 5 kg). These space allowances were applied in accordance with the Canadian Transport Codes recommendation [14] for the transport of pigs of these body weights in periods of the year other than those hot and humid. The real average final body weight of pigs selected for the study was 120.4 kg and 140.5 kg for the lighter and heavier weight groups, respectively. Similarly to the summer study, space allowance × slaughter weight combinations were rotated across the four compartments in each shipment or replicate (Table 2).

On the day prior to each shipment, 24 focal immunocastrated boars (six/truck compartment or space allowance × market weight combination), totalling 96 focal immunocastrated boars through the whole experiment, were selected from different finishing pens, weighed, and tagged in both ears for identification during blood sampling at slaughter and carcass meat quality measurements, and were returned to the finishing pen where they were sorted out from during loading. In preparation for transport, feed was withdrawn from all pigs for approximately 7 h before loading (approx. 11 h total fasting time at slaughter).

On the day of transportation, pigs, including focal ones, were loaded in small mixed groups of five to ten pigs using paddles and sorting boards only. The truck left the farm upon completion of the loading process (wait-at-farm: 10 min on average, ranging from 5 to 19 min). During transport, only 10% of the side panels were open to protect pigs from cold while providing sufficient airflow for air exchange in the truck. The truck was also bedded with fresh wood shavings. Two drivers were rotated across shipments or replicates to randomise the effect of the driver on handling and driving on pigs’ response to transport [12].

Pigs were unloaded almost immediately upon arrival at the abattoir (wait-at-abattoir: 4 min on average, ranging from 2 to 9 min) in groups of 13 pigs, which included six focal pigs, using paddles, and were driven to separate lairage pens based on the treatment combination or truck compartment. No mixing of pigs between compartments/treatment combinations occurred. As the size of truck compartments and lairage pens was different, to keep a constant stocking density (0.70 m^2^/pig) in the lairage pen, the group size was reduced to 13 pigs/group (including the six focal pigs) in each lairage pen. Pigs were kept in lairage for 2.5 h on average, with free access to water.

After lairage, pigs were driven using paddles along the alleys and an automatic push gate system in the last chute feeding the CO_2_ gas stunner (Frontmatec AS, M7-RR-S1, Kolding, Denmark). Immediately upon exiting the stunner, pigs were shackled and exsanguinated in the vertical position.

### 2.3. Data Collection (Both Studies)

#### Ambient Climate and Trailer Microclimate Measurements

Ambient air temperature and humidity data were collected using iButton data loggers (DS1923 Hydrochron Temperature/Relative Humidity Logger, Maxim Integrated Products Inc., Sunnyvale, CA, USA) attached on the inside of the four compartments (five iButtons/compartment). The iButtons were placed approximately 8 cm from the ceiling, with one positioned in the centre of the compartment and the other four placed in each corner of the compartment. The iButtons were programmed to record temperature (T) and relative humidity (RH) data every minute from the beginning of loading to the end of unloading. The temperature range of the data logger was from −20 to +85 °C with a resolution of ±0.0625 °C and an accuracy of 0.5 °C, and a relative humidity range from 0 to 100% with a resolution of ±0.04% and an accuracy of 5%. The iButtons were programmed and data were downloaded after each transport using the ExpressThermo software (ECLO Solutions, Leiria, Portugal).

### 2.4. Behaviour Observations

Behaviour during lairage was recorded by a video camera (HDRAS100 V, Sony Corp., Tokyo, Japan) installed on each of the lairage pen walls to survey the pigs. The recording started as soon as the pen was filled with pigs and the gate was closed.

All treatment groups were observed for a minimum time of 72 min. Scan sampling (every 2 min) was used to record the number of pigs lying down, standing, sitting, or engaging in other postures, while a continuous sampling was performed to record drinking and fighting behaviours (Table 3).

For the postural behaviour, the average percentage of pigs standing, lying, sitting, and other behaviours was calculated. Due to its low percentage during lairage, sitting behaviour data were merged with other postures. Data were analysed for the overall 72 min and separately for the first and second half of the lairage observation time (36 min each). The latency to lie down was calculated as the time (min) from the start of lairage until at least 45% of pigs were lying down, which was the minimum proportion reached by all compartment groups within the observation period (72 min) in this study.

Drinking behaviour during lairage was analysed for the number of drinking bouts, percentage of time spent drinking, and latency for the first pig to drink by treatment group. Fighting behaviour was analysed for the percentage of time spent fighting, the total number of fights, and latency for the first pig to fight by treatment group. Data for drinking and fighting behaviour were analysed for the total 72 min observation time in lairage.

Behavioural observations were performed by one trained observer using The Observer XT software (version 15; Noldus Information Technology Inc., Wageningen, The Netherlands), and the average correlation coefficient for intra-observer agreement for all behaviours observed was 0.99.

### 2.5. Blood Sampling and Analysis

At exsanguination, blood was collected from the bleeding wound of the 24 focal pigs per replication (a total of 144 pigs during summer and 96 pigs during winter) in serum tubes (BD Vacutainers^®^, VWR International Ltd., Montreal, QC, Canada). Whole-blood lactate concentrations were immediately assessed in duplicate with a hand-held Lactate Scout Analyzer (Lactate Scout, EKF Diagnostic GmbH, Magdeburg, Germany) by dipping a test strip (two strips/animal) into a serum tube containing the collected blood. Another blood sample was also collected in a second serum tube for CK analysis. Serum was collected after centrifugation at 1400× *g* for 10 min at 4 °C and then stored at −80 °C until analysis. Serum CK concentration was analysed using a creatine kinase_SL kit (Creatine Kinase-SL Assay, SEKISUI Diagnostics, Charlottetown, PE, Canada) and determined with a spectrophotometer. The intra-assay coefficient of variation for log-transformed blood CK was 2.70% in summer blood samples and 2.58% in winter blood samples. A third blood sample was collected in an EDTA tube (BD Vacutainers^®^; VWR International Ltd., Montreal, QC, Canada), refrigerated at 4 °C and subsequently analysed in duplicate for haematocrit determination according to the microhematocrit procedure described by Matte et al. [35].

### 2.6. Carcass and Meat Quality Measures

Carcasses were dehaired, singed, eviscerated, split, and chilled according to the standard operating procedures of the abattoir.

Hot carcass weight (HCW) was obtained from the grading slips, and carcass yield (%) was calculated. Skin damage was assessed on the whole carcass in the cooler by a single observer using a subjective five-point photographic scale from 0 = no to very minimal lesions to 5 = severe lesions [36].

All meat quality measurements were taken at 24 h *post-mortem* in the *longissimus* muscle (LM), between the third and fourth last rib, and in the *semimembranosus* (SM) and *adductor* (AD) muscles. The pHu was measured using a portable pH-metre (Oakton Instruments Model pH 450 series, Nilis, IL, USA) fitted with a spear tip pH electrode (Cole Palmer Canada, Montreal, QC, Canada) and an automatic temperature compensation (ATC) probe (Oakton Instruments, Vernon Hills, IL, USA).

Instrumental colour (L*, a*, b*; [37]) was measured with a CM700d Spectrophotometer (Konica Minolta Sensing Inc., Osaka, Japan) equipped with an 8-mm aperture, 10° viewing angle, and D65 illuminant after exposing the muscles LM and SM surface to 15 min blooming time.

Meat exudation was measured in the LM (same location as pHu measurement) using the filter paper wetness (FPW) test described by Kauffman et al. [38]. Briefly, a pre-weighed filter paper (Whatman PK100, VWR International Co., Mont-Royal, QC, Canada) was placed on the LM cut surface after 10 min of air exposure and reweighed using an analytical scale (Scout SPX, OHAUS, Parsippany, NJ, USA) after 3 s of fluid accumulation on the paper. The drip loss percentage was calculated by the following equation: [% drip loss = −0.1 + (0.06 × mg fluid)] [10]. In the summer study, drip loss was measured in the SM muscles using a modified EZ-driploss method [39]. Briefly, two 25 mm diameter muscle cores were removed from the SM muscle, weighed, and placed into plastic drip loss containers (KABE Labortechnik, Umbrecht-Elsenroth, Germany) and stored for 48 h at 4 °C. At the end of the 48 h storage period, the surface moisture of the muscle cores samples was carefully dabbed, and the cores were reweighed. The drip loss percentage was calculated by dividing the difference between the initial and final core weights by the initial core weight. Whereas, in the winter study, exudation in the SM muscle was measured by electrical conductivity using a Pork Quality Metre equipped with a conductivity probe (CLASSPRO GmbH, Aichach, Germany).

### 2.7. Calculations and Statistical Analysis

In both seasons, within-truck average T and RH values were calculated for each transport phase (i.e., loading, wait-at-farm, transport, and wait-at-abattoir; Table 4) by averaging the five iButton logger data per compartment, of which the average values were calculated over the whole time of each phase.

The analysis was performed according to a 2 × 2 factorial design using replicates as random blocks within the analytical model and the compartment as the experimental unit. All truck microclimate, pig physiological, lairage behaviour, and meat quality data analyses were performed using SAS software (version 9.4; SAS Inst. Inc., Cary, NC, USA), where analysis was performed considering space allowance, slaughter weight, and their interaction as fixed factors in a 2 × 2 factorial design. Results are presented as least-squares means (LSM) ± SEM. Replicate was considered a random factor.

In the summer study, temperature humidity index (THI), which is normally used as an indicator of ambient conditions in heat stress studies and livestock transport guidelines [40,41], was calculated based on the T° and RH values according to the formula: THI = (1.8 × T + 32) − [(0.55 − 0.0055 × RH) × ((1.8 × T − 26)], where T is in °C and RH in % [41]. To provide a more complete description of the within-truck air quality, the enthalpy of air (h), indicative of the thermal comfort index that expresses the heat energy of the air (1 kg dry air in kJ) surrounding an animal, and dictates the degree of heat loss to the microclimate, was also calculated by the equation: h = (6.7 + 0.243 × T + ((RH/100) × 10(7.5 × T)/(237.3 + T)) × 4.18, where T is in °C and RH in % [42].

For physiological and meat quality data analyses, the group of six sentinel pigs per compartment was considered as the experimental unit. Basic ANOVA assumptions were verified using the studentised residuals. Usual correcting steps were applied, if necessary. A probability level of *p* ≤ 0.05 was chosen as the limit for statistical significance in all tests. Observed probabilities of *p* ≤ 0.10 were considered tendencies.

As a result of unforeseen events, during summer, lairage behaviour data related to the 0.54 m^2^/120 kg treatment group were missing due to one camera recording failure. Furthermore, in total (summer and winter studies combined), 12 carcasses (6% and 6.2% in summer and winter, respectively) were lost through the slaughter chain. Owing to the difficulty of keeping pace with the speed of the bleed line on the kill floor, or due to poor sample quality, a total of 21 blood lactate (14% and 1% in summer and winter, respectively), 14 blood haematocrit (9% and 1% in summer and winter, respectively), and 16 CK (10% and 2% in summer and winter, respectively) samples were lost. Furthermore, it was not possible to assess the lesion score in a total of 8 carcasses (3% in both seasons) and record 13 HCW (8% and 1% in summer and winter, respectively).

## 3. Results

Due to differences in pig genetics, farm of origin, and transport conditions between the summer and winter studies, results cannot be compared between seasons and are thus presented separately.

### 3.1. Summer Transports

#### Within-Truck Microclimate

As shown in Table 5, within-truck microclimate characteristics were neither influenced by space allowance, slaughter weight, nor their interaction (*p* > 0.10).

### 3.2. Behavioural Observations

The interaction of space allowance × slaughter weight did not affect postures during lairage (*p* > 0.10).

**Figure 3:**
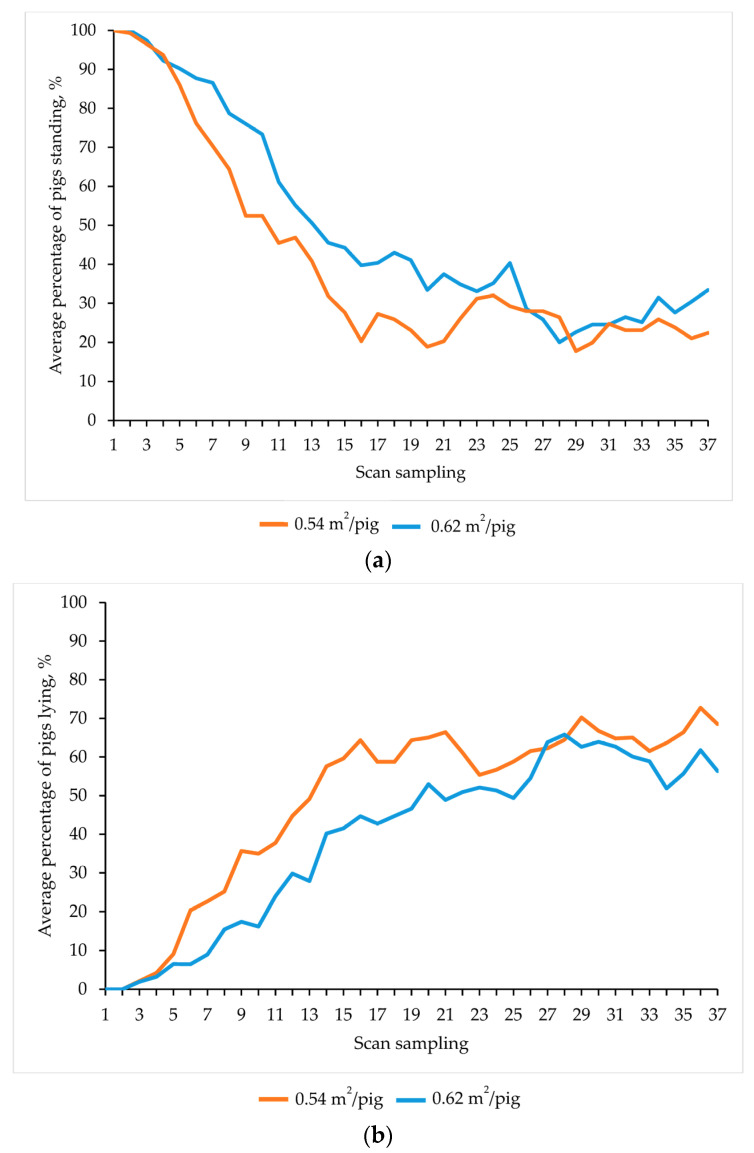
Effects of space allowance on the percentage of pigs standing (**a**) and lying (**b**) during the 72 min of lairage observation time (scans every 2 min) in summer.

For the entire observation period (72 min), space allowance tended to affect postures, where a greater proportion of pigs that were provided with greater space allowance in the truck (0.62 m^2^/pig) stood more and lay less in the lairage pen (*p* = 0.07 and *p* = 0.06, respectively; Figure 3a,b) than pigs transported at lower space allowance (0.54 m^2^/pig). However, this effect was significant in the first half of the observation time (36 min), where pigs transported at a greater space allowance (0.62 m^2^/pig) stood in greater percentage (*p* = 0.04) and, consequently, lay down in lower percentage (*p* = 0.02) compared to pigs transported at a smaller space allowance (0.54 m^2^/pig). Space allowance neither had any impact on the behaviours observed during the second half of the observation time in lairage (*p* > 0.10) nor on the proportion of pigs in other postures during the whole observation time (*p* > 0.10). Slaughter weight did not affect the percentage of pigs in any posture during lairage (*p* > 0.10). Neither space allowance nor slaughter weight affected the latency to lie (*p* > 0.10).

As shown in Table 6, the interaction of space allowance × slaughter weight had no effect on drinking and fighting behaviours (*p* > 0.10). When compared to pigs transported at a greater space allowance (0.62 m^2^/pig), pigs transported at the lower space allowance (0.54 m^2^/pig), started fighting later (25.07 ± 4.61 vs. 11.20 ± 4.40 min.; *p* = 0.04), and tended to fight less (7.63 ± 3.34 vs. 15.83 ± 3.19 fights; *p* = 0.09).

Slaughter weight also had an effect on drinking during lairage, with heavier pigs having a longer latency to start drinking (171.7 [105.6–279.1] vs. 75.4 [45.3–125.5] s; *p* = 0.02) and having fewer drinking bouts (31.2 ± 9.57 vs. 57.3 ± 9.79; *p* = 0.05) than lighter pigs.

Slaughter weight also had an effect on fighting behaviour during lairage, with heavier pigs spending a greater percentage of time fighting (11.7 ± 3.19 vs. 2.4 ± 3.32%; *p* = 0.05) and fighting more (17.3 ± 3.19 vs. 6.1 ± 3.34 fights; *p* = 0.03) than lighter pigs.

### 3.3. Blood Variables

As shown in Table 7, the interaction of space allowance × slaughter weight had an effect on blood haematocrit levels (*p* = 0.04), with higher blood haematocrit levels being found at slaughter in heavier pigs when transported at a smaller space allowance (0.54 m^2^/pig) compared to higher space allowance (0.62 m^2^/pig) (*p* < 0.02).

Blood lactate concentration at slaughter was neither influenced by space allowance, slaughter weight, nor their interaction (*p* > 0.10). Meanwhile, slaughter weight affected serum CK concentrations at slaughter with greater CK levels found in heavier pigs compared to lighter pigs (3.95 ± 0.05 vs. 3.77 ± 0.04 UI/L; *p* < 0.01).

### 3.4. Carcass and Meat Quality

Neither space allowance, slaughter weight, nor their interaction influenced carcass yield or lesion scores (*p* > 0.10; Table 8). Slaughter weight affected hot carcass weight, with carcasses from heavier pigs being heavier than carcasses from lighter pigs (115.6 ± 0.65 vs. 100.3 ± 0.65; *p* < 0.001).

Space allowance had an effect on Minolta b* value, with loins tending to be yellower (10.40 ± 0.34 vs. 11.05 ± 0.34; *p* = 0.07) and SM muscle being yellower (9.85 ± 0.17 vs. 9.97 ± 0.17; *p* < 0.01) in pigs transported at a greater space allowance. When compared to lighter pigs, heavier pigs presented a slightly drier (lower drip loss) SM muscle (2.85 ± 0.18 vs. 3.34 ± 0.18%; *p* = 0.05) and a trend for a higher pHu value in the AD muscle (5.93 ± 0.07 vs. 5.82 ± 0.07; *p* = 0.07).

### 3.5. Winter Transports

#### Within-Truck Microclimate

As shown in Table 9, within-truck microclimate characteristics were neither influenced by space allowance, slaughter weight, nor their interaction (*p* > 0.10).

### 3.6. Behavioural Observations

**Figure 4:**
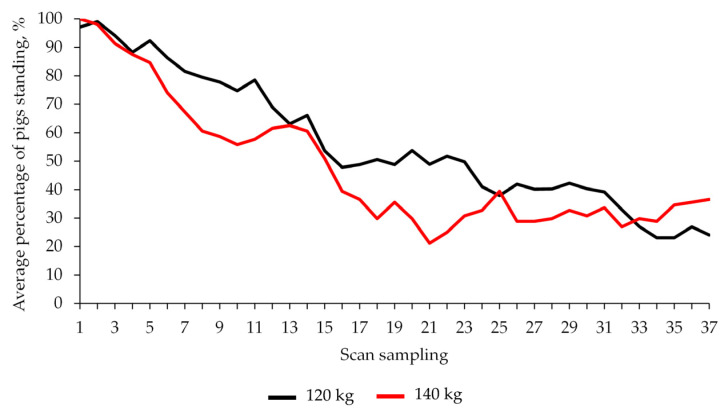
Effects of slaughter weight on the percentage of pigs standing during lairage (scans every 2 min over 72 min observation time) in winter.

A greater proportion of heavier pigs tended to stand less during the first half and the whole observation time in lairage when compared with lighter pigs (*p* = 0.08 and *p* = 0.07 for each period, respectively; Figure 4), although the expression of this posture decreased over time in both pig types. However, this posture was not associated with a variation in lying behaviour, which did not differ between the two groups of pigs (*p* > 0.10).

Space allowance had no effect on postural behaviour during lairage (*p* > 0.10). Furthermore, neither space allowance nor slaughter weight affected the latency to lie down (*p* > 0.10).

**Figure 5:**
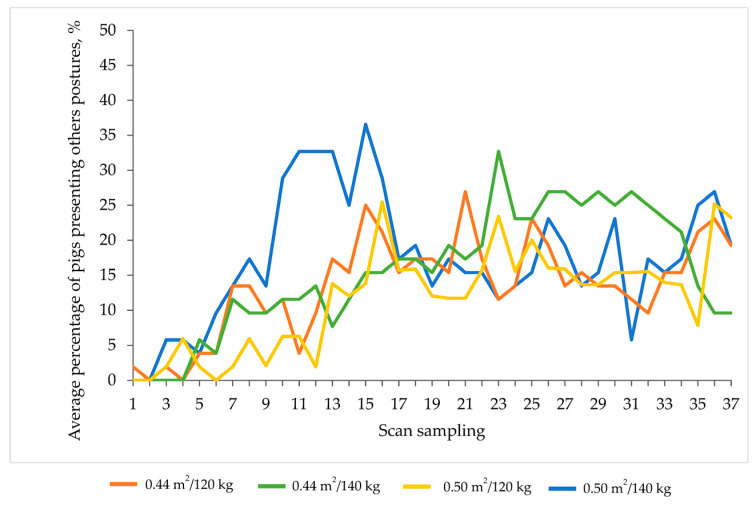
Interaction between space allowance and slaughter weight on the percentage of pigs sitting or presenting other postures during lairage (scans every 2 min over 72 min observation time) in winter.

The interaction space allowance × slaughter weight tended to affect the expression of sitting or other postures in lairage (*p* = 0.08; Figure 5), with a greater proportion of 140 kg pigs transported at 0.50 m^2^/pig sitting or showing other postures compared to lighter pigs transported at the same density during the first half of the observation time (36 min) in lairage.

No effects of slaughter weight, space allowance, or their interaction were observed for drinking or fighting behaviours (*p* > 0.10; Table 10).

### 3.7. Blood Variables

As shown in Table 11, neither space allowance, slaughter weight, nor their interaction influenced blood haematocrit, lactate and CK concentrations (*p* > 0.10).

### 3.8. Carcass and Meat Quality

Neither space allowance, slaughter weight, nor their interaction influenced carcass yield or lesion scores (*p* > 0.10; Table 12). Slaughter weight had an effect on hot carcass weight, with carcasses from heavier pigs being heavier than carcasses from lighter pigs (113.1 ± 0.69 kg vs. 96.4 ± 0.69 kg; *p* < 0.001).

Space allowance influenced the pHu value in the LM, with slightly higher pHu being found in pigs transported at 0.44 m^2^/pig compared with 0.50 m^2^/pig (5.77 ± 0.07 vs. 5.73 ± 0.07; *p* = 0.03). When compared to lighter pigs, in the heavier pigs the pHu value tended to be slightly lower in the LM muscle (5.74 ± 0.07 vs. 5.77 ± 0.07; *p* = 0.10) and was slightly lower in the SM muscle (5.75 ± 0.03 vs. 5.82 ± 0.03; *p* = 0.05). The L* value of the SM muscle of heavier pigs was also slightly higher, indicative of a paler colour, compared with lighter pigs (48.51 ± 0.27 vs. 47. 59 ± 0.27; *p* = 0.03).

## 4. Discussion

The thermal environment inside the trailer is considered a serious concern for the health and welfare of pigs during transport [43]. Since pigs are homoeothermic animals, the surrounding environment should ensure their balance between heat production and heat loss [44]. Heat stress is particularly concerning in the case of heavier-weight pigs, which are more vulnerable to heat stress than lighter-weight pigs [17] as they produce more body heat (+2% per 5 kg body weight increase) and are less capable of dissipating it [16]. Reduced floor space in the trailer, resulting in overcrowding, or poorly ventilated environments, like those registered in passively ventilated transport vehicles [30,31,45], may worsen the effects of heat stress in these pigs. Dewey et al. [46] reported a 7 °C increased temperature inside a passively ventilated trailer by adding only two extra pigs per m^2^ in the compartment. This thermal discomfort may explain the increased risk of animal losses in pigs transported at greater load density or size [13,47].

Overall, during summer, except for the wait-at-farm phase, the temperature inside the test compartments was within the upper threshold of the thermo-neutral zone for market-weight pigs (22 °C; [48]). The significant increase in internal temperature inside a stationary passively ventilated vehicle has been extensively reported [49]. However, the temperature increase recorded in the wait-at-farm phase in this study was below the threshold for upper critical temperature (UCT = 25 °C; [48]), which is defined as the point above which an animal must significantly increase the use of physiological mechanisms to prevent a rise in body temperature above normal [50]. The mild external temperature jointly with the short stationary phase might have helped keep pigs in their thermal comfort zone. Similarly, within-truck RH and THI did not exceed the thresholds of 88% and 75, respectively, which are defined as critical values as they may significantly compromise the pigs’ evaporative cooling mechanisms, such as reduced activity, accelerated breathing and contact with cool and wet surfaces, which may result in death [44,51]. However, the overall value of enthalpy of the air (h = 63.34) was between the proposed thresholds for mild and severe heat stress (h = 55–65) in growing-finishing pigs [52]) across all transport events, which may indicate a potential risk for thermal discomfort, regardless of the external ambient and transport conditions applied in this study. The difference in the THI and enthalpy results confirms the discrepancy between these two measures of ambient parameters that was explained by the lack of accuracy of the THI measurement not considering, among other factors, the effect of airflow and ventilation, and animal differences, e.g., body weight [48].

Overall, during winter, the temperature inside the test compartments was below the threshold of the lower critical temperature (LCT) of the thermoneutral zone, which is defined as the point in effective ambient temperature below which an animal must increase its rate of metabolic heat production to maintain homeothermy and that for the transport of 100 kg to 140 kg market-weight pigs ranges from 10 °C to 14 °C [53]. These recommended LCT thresholds can be reduced by 3 °C to 5 °C in the case of well-bedded floors [54], as those used in this study, but, even so, during all transport phases of this study pigs were likely in some thermal discomfort. Leaving the two middle compartments empty must have contributed to reducing the quality of the ambient conditions inside the truck as the heat load generated by the pigs must have been lower compared with a fully loaded truck.

The behaviour of pigs in lairage can be explained by their physical state on arrival at the abattoir, which, in turn, is influenced by their behavioural response during transport [12]. Urrea et al. [55] reported a 10-fold lower drinking bout frequency during lairage in pigs transported at greater loading density (270 vs. 200 kg/m^2^) and associated this behaviour with greater fatigue-related resting during lairage. However, the different space allowances during summer and winter transports had no effect on the number of drinking bouts and time spent drinking, but, similarly to Urrea et al. [55], a higher proportion of pigs transported at a smaller space allowance (240 vs. 209 kg/m^2^) in the truck during summer preferred resting during the first half of lairage observation time, which delayed activities, such as fighting unfamiliar pen-mates, compared to pigs transported at greater space allowance. Although it was not possible to make reliable on-truck behaviour observations due to poor recording conditions in this study (low deck ceiling and standing pigs blocking the view of the rest of the compartment group), based on the observations from previous studies [49,55], the fatigue condition of these pigs upon arrival in the lairage pen may be explained by the inability of the whole compartment load to lie down simultaneously during the journey, which resulted in a continual change of position, preventing resting [49]. In summer, heavier pigs spent the greatest proportion of lairage observation time (72 min) fighting unfamiliar pen-mates, which affected their fitness at slaughter (greater serum CK levels at slaughter). The consequential delayed drinking activity in heavier pigs might have contributed to increasing their dehydration status (higher blood haematocrit level) at slaughter, especially when transported at a smaller space allowance as a consequence of heat stress. The increased aggressiveness seen in mixed groups of heavier pigs in lairage confirms the correlation between body weight and conformation, and agonistic behaviour reported in a study on piglets [56].

Signs of some sort of physical fatigue condition on arrival at the abattoir have been also recorded after winter transports, especially in heavier pigs provided with a larger space allowance in the truck, that tended to sit or kneel, among other postures, during rest in lairage. Kneeling and sitting are, in fact, defined as transition postures from standing to lying with some indication of fatigue as pigs spent a few seconds in these postures before lying down [57,58]. The fatigue condition of heavier pigs on arrival at the abattoir may have resulted from the lack of time to recover from loading stress during the short transport (1 h) and the greater effort to keep a standing posture during transit, which is the dominant posture in transports shorter than 2–3 h [49]. It has been reported that the provision of larger space in the truck during short journeys (≤1 h) would be even more detrimental to the welfare of pigs than tighter space as it prevents standing or sitting pigs from keeping their balance by holding each other while the vehicle negotiates turns or rough road surfaces, which may result in injuries or bruises and poor meat quality [26,59]. According to Noblet et al. [60], standing requires two times more energy than lying down, which leads to greater physical exhaustion. However, similarly to the summer transports, the poor recording conditions (low deck ceiling combined with standing pigs blocking the view of the rest of the compartment group) did not allow for the analysis of in-transit behaviour and limited the interpretation of lairage behaviours recorded during this season.

The above-mentioned signs of fatigue observed in pigs during winter lairage did not translate into a difference in the concentration of stress variables analysed in exsanguination blood. However, similarly to summer, the blood lactate concentrations revealed a general fatigued condition at slaughter in pigs transported during this season, regardless of the journey conditions and slaughter weight, as they were well above the reported whole blood rest concentrations (3.5 to 4 mmol/L; [61]) and the threshold of 7 mmol/L, which has been associated to increased risk for abnormal *post-mortem* meat acidification [62]. This result is not surprising considering that even in a minimal-stress situation, like that in this study (sufficient rest time plus group-wise handling using push-gates to the stunner), blood lactate concentration increases [63].

Serum CK concentration is a good indicator of long-term muscular activity or tissue damage in pigs at slaughter [49]. The lack of effect of space allowance on blood CK concentrations at slaughter in both studies is in contrast with the results of previous studies that reported higher serum CK concentrations in pigs transported at high density [55,59]. An explanation for the lack of effects may be the short-term effect of transport applied prior to slaughter in these studies considering the half-life of CK activity in the blood (peak at 6 h after physical stress exposure [49]). The similarity in fighting behaviour during rest may also explain the lack of difference in blood CK levels between lighter and heavier pigs during winter.

Overall, the recorded skin lesion scores were within the range of the slight lesion class in both summer and winter loads [36]. However, in neither study lesion scores were influenced by space allowance, slaughter weight, or their interaction, despite the effects of these factors on the fighting rate in the lairage pen in summer. Fighting-type lesions are the most frequently observed lesions on the pig carcass at slaughter [64]. During both summer and winter transports, floor space allowance did not affect skin lesion scores, which conflicts with several previous studies [55,59,65], where, however, a tighter floor space (<0.42 m^2^/pig) was provided to pigs during transport.

A recent study reported a higher proportion of severe carcass lesions in heavier-weight pigs (133 vs. 85 kg on average; [66]), whereas similar to our studies, Rocha et al. [10] failed to find differences in skin bruise scores between pigs of two different slaughter weights, although the groups only differed by 13 kg in their study. The discrepancy in the results between studies may be thus explained by the body weight difference between tested groups.

Based on existing literature reports, both the provision of greater and lower floor space can result in muscle fatigue, as shown by the higher pHu values, an indicator of DFD (dark, firm, dry) pork meat, due to muscle glycogen depletion [27,49]. In both summer and winter studies, the major effects were found in the ham muscles, which is not surprising as SM and AD are locomotor muscles and are thus more prone to rapid glycogen exhaustion after physical exercise rather than postural muscles, such as the LM [67]. However, results were clearer in summer, where heavier pigs presented a drier SM muscle and a tendentially higher pHu in the AD muscle, both traits likely associated with their greater fighting activity during lairage and resulting fatigue condition of these pigs at slaughter (as shown by the higher CK level in exsanguination blood). During winter, space allowance and slaughter weight influenced pHu and Minolta L* values in the loin and ham muscles, but the effects were minor and hard to explain based on the lack of effects of these factors on the within-truck ambient and animal-based parameters assessed in this study. Overall, the differences in these meat quality traits in both studies are of little, if none, biological and economic significance.

## 5. Conclusions

Based on the results obtained under the transport conditions of these studies, the effects of providing space allowances of 0.54 or 0.62 m^2^/pig during summer on within-truck ambient conditions, pigs’ post-transport animal welfare, and carcass and meat quality are similar, regardless of the slaughter weight.

The summer results may confirm the relationship between reduced floor space to lie down under warmer transport conditions and the pigs’ fatigue condition on arrival at the abattoir. This physiological condition increased pig resting by reducing the time for fighting during lairage and fatigue at slaughter.

No clear conclusions can be drawn based on the results of the winter study that were biased by the short journey and the condition of the truck not travelling at its full load capacity.

Further research is needed to validate the space allowances applied in both studies for heavier pigs under longer transport conditions (>3 h) since pigs are more likely to stay standing during the first 2–3 h of the journey and cannot take advantage of the floor space provided to rest and recover after loading, and better cope with warm and cold temperature conditions.

The results of the summer study highlight the effects of increased slaughter weight on the behaviour of pigs, which, alongside being more difficult to handle as reported in previous studies, are more aggressive under mixed group conditions during lairage, resulting in fatigue at slaughter and a greater risk of DFD-like pork meat production.

## Figures and Tables

**Table 1:** Distribution of load size (n of pigs), real applied space allowances (SA), and slaughter weights (SW) across truck compartments (C) through the replicates during summer.

	C1			C2			C3			C4		
Replicate	n	SW	SA	n	SW	SA	n	SW	SA	n	SW	SA
1	17	121.3	0.54	13	125.2	0.61	15	139.3	0.63	15	142.8	0.54
2	15	122.5	0.61	15	143.2	0.53	17	123.9	0.56	13	141.5	0.62
3	17	142.8	0.54	13	141.5	0.61	15	122.7	0.63	15	124.2	0.54
4	14	142.4	0.65	16	125.6	0.50	17	144.3	0.56	13	124.0	0.62
5	17	124.6	0.54	13	124.3	0.61	15	140.0	0.63	15	142.4	0.54
6	15	122.8	0.61	15	145.8	0.53	17	123.3	0.56	12	141.8	0.67

**Table 2:** Distribution of load size (n of pigs), space allowances (SA), and slaughter weight (SW) across truck compartments (C) through the replicates during winter.

	C1			C2			C3			C4		
Replicate	n	SW	SA	n	SW	SA	n	SW	SA	n	SW	SA
1	16	122.3	0.50	21	139.0	0.42	20	121.5	0.44	18	138.7	0.50
2	18	141.7	0.44	19	140.9	0.47	17	120.6	0.51	21	121.4	0.43
3	15	141.7	0.53	21	121.3	0.42	19	141.4	0.46	18	121.6	0.50
4	18	117.2	0.42	19	119.5	0.49	16	140.7	0.55	19	140.9	0.47

**Table 3:** Description of behaviours evaluated during lairage in both seasons.

Behaviour	Description
Standing	The pig is supporting itself in an upright position using three or four extended legs where the belly is not in contact with the ground.
Lying	The pig is not using any limbs to support itself and may either have full belly contact with the ground or another pig or be lying on its sides with that side touching the ground or another lying pig.
Sitting	The pig is supporting itself with one or two of its forelimbs where the hindlimbs are not supporting the pig’s body and the rear of the pig is making contact with the ground or another pig that is lying down.
Other posture	The pig is not in a previously defined position, as when a pig was neither standing, sitting, or lying, such as kneeling (the hindlimbs are engaged and the forelimbs are not supporting the pig’s body).
Drinking bouts	The pig puts its mouth around the drinker for any period of time (a new bout is recorded if the pig’s mouth is off the drinker for at least 5 s).
Fighting	Behaviour includes “biting” and/or “violent head-knock”, which can also include “pushing with the whole body or shoulder” or when one pig is confronting another pig with “biting” and/or “rapid violent head-knock”. The sequence should last at least 2 s without a break longer than 10 s. A break longer than 10 s was considered a new fight. If a third pig entered the fight, a new observation was counted.

**Table 4:** Definition of the experimental transport phases during both seasons.

Phases	Definition
Loading	Period of time between the first pig of the treatment group entering the truck compartment and the last pig of the group entering the truck compartment, and the compartment gate was closed.
Wait-at-farm	Period of time from the end of the loading phase (or truck door was closed) until the truck departed from the farm.
Transport	Period of time between the truck’s departure from the farm and the truck’s arrival at the abattoir.
Wait-at-abattoir	Period of time between the truck’s arrival and stops at the abattoir and the start of unloading, i.e., the truck door opened to allow pigs to exit the truck.

**Table 5:** Effects of space allowance (SA; 0.54 and 0.62 m^2^/pig), slaughter weight (SW; 120 kg and 140 kg), and their interaction on average temperature (T), relative humidity (RH), temperature humidity index (THI) ^1^, and enthalpy (h) ^2^ inside the truck compartments during summer.

Variable	Treatment							
	0.54 m^2^/pig		0.62 m^2^/pig			*p*-Value		
	120 kg	140 kg	120 kg	140 kg	SEM	SA	SW	SA × SW
Loading								
T, °C	19.96	20.15	20.01	20.06	0.61	0.79	1.00	0.88
RH, %	84.66	86.77	86.63	84.30	4.43	0.93	0.86	0.14
THI	67.19	67.52	67.28	67.30	1.11	0.79	0.92	0.82
h	61.95	62.51	62.22	62.05	1.53	0.77	0.88	0.59
Wait-at-farm								
T, °C	24.25	24.16	23.89	24.03	0.40	0.90	0.25	0.59
RH, %	80.26	81.70	82.39	81.34	4.41	0.86	0.46	0.30
THI	73.69	73.74	73.35	73.48	0.93	0.74	0.30	0.88
h	69.33	69.55	69.06	69.13	1.50	0.69	0.35	0.83
Transport								
T, °C	20.01	19.85	19.87	20.00	0.88	0.97	0.98	0.59
RH, %	83.05	83.97	84.00	83.64	3.80	0.78	0.76	0.53
THI	67.08	66.86	66.89	67.12	1.54	0.98	0.93	0.58
h	61.76	61.57	61.64	61.81	1.90	0.96	0.86	0.60
Wait-at-abattoir								
T, °C	21.72	21.44	21.33	21.32	0.60	0.65	0.42	0.66
RH, %	80.45	82.20	81.79	81.71	4.22	0.41	0.67	0.37
THI	69.76	69.35	69.23	69.15	1.13	0.59	0.42	0.71
h	64.51	64.18	64.01	63.90	1.61	0.63	0.38	0.80

^1^ THI = (1.8 × T + 32) − [(0.55 − 0.0055 × RH) × (1.8 × T − 26)] [40]. ^2^ h: Enthalpy (kJ/kg dry air) = (6.7 + 0.243 × T + ((RH/100) × 10(7.5 × T)/(237.3 + T)) × 4.18 [41].

**Table 6:** Effects of space allowance (SA; 0.54 and 0.62 m^2^/pig), slaughter weight (SW; 120 kg and 140 kg) and their interaction on drinking and fighting behaviour during lairage in summer.

Variable	Treatment							
	0.54 m^2^/pig		0.62 m^2^/pig			*p*-Value		
	120 kg	140 kg	120 kg	140 kg	SEM	SA	SW	SA × SW
Drinking								
Latency to drink ^a^, s	131 [63–273]	173 [89–339]	43 [22–85]	170 [87–332]		0.10	0.02	0.11
Time spent drinking, %	18. 8	12.2	18.9	14.7	5.92	0.82	0.34	0.83
Drinking bouts, n	52.6	32.3	56.7	30.2	12.02	0.93	0.05	0.78
Fighting								
Latency to fighting, min	30.8	19.38	12.2	10.2	6.77	0.04	0.30	0.46
Time spent fighting, %	0.6	9.8	4.4	13. 6	4.66	0.40	0.05	0.99
Total fights, n	2.6	12.7	9.7	22.0	4.94	0.09	0.03	0.81

^a^ Data were log-transformed, and values are reported as back-transformed least-square mean with the limits of the confidence interval in square brackets.

**Table 7:** Effects of space allowance (SA; 0.54 and 0.62 m^2^/pig), slaughter weight (SW; 120 kg and 140 kg) and their interaction on average blood haematocrit, lactate, and creatine kinase (CK) concentrations at slaughter during summer.

Variable	Treatment							
	0.54 m^2^/pig		0.62 m^2^/pig			*p*-Value		
	120 kg	140 kg	120 kg	140 kg	SEM	SA	SW	SA × SW
Haematocrit, %	27.8	32. 9	29.8	24.8	2.90	0.18	0.99	0.04
Lactate, mmol/L	9.8	10.9	9.87	10.19	0.76	0.65	0.35	0.62
CK, log UI/L	3.72	3.97	3.82	3.94	0.06	0.57	<0.01	0.25

**Table 8:** Effects of space allowance (SA; 0.54 and 0.62 m^2^/pig), slaughter weight (SW; 120 kg and 140 kg), and their interaction on carcass lesion scores and pork quality traits as assessed in the *longissimus* muscle (LM), *semimembranosus* (SM), and *adductor* (AD) muscles during summer.

Variable	Treatment							
	0.54 m^2^/pig		0.62 m^2^/pig			*p*-Value		
	120 kg	140 kg	120 kg	140 kg	SEM	SA	SW	SA × SW
HCW, kg ^1^	100.7	116.5	99.9	114.6	0.77	0.03	<0.001	0.34
Carcass yield, %	81.1	81.2	81.2	81.1	0.42	0.87	0.85	0.65
Lesion score ^2^	1.60	1.56	1.41	1.67	0.18	0.79	0.48	0.31
LM								
pHu	5.70	5.75	5.64	5.70	0.06	0.22	0.23	0.81
L*	50.08	50.15	50.98	50.66	0.72	0.16	0.79	0.67
a*	3.12	3.14	3.64	3.39	0.27	0.11	0.62	0.55
b*	10.40	10.49	11.12	10.97	0.41	0.07	0.83	0.81
Drip loss, % ^3^	1.49	1.59	1.49	1.50	0.19	0.82	0.77	0.85
SM muscle								
pHu	5.80	5.73	5.78	5.81	0.04	0.48	0.58	0.19
L*	46.73	46.41	46.61	47.02	0.59	0.66	0.94	0.52
a*	3.64	3.35	3.92	3.64	0.23	0.12	0.13	0.96
b*	9.91	9.80	10.32	10.36	0.19	<0.01	0.78	0.49
EZ-Drip Loss	3.36	3.12	3.32	2.58	0.24	0.22	0.05	0.28
AD muscle								
pHu	5.82	5.86	5.83	5.99	0.08	0.22	0.07	0.29

^1^ HCW: Hot carcass weight. ^2^ Based on photographic charts (from 1: none to 5: severe; [35]). ^3^ Percentage of drip loss was calculated by the equation: [% drip loss = −0.1 + (0.06 × mg fluid)] [10].

**Table 9:** Effects of space allowance (SA; 0.44 m^2^/pig and 0.50 m^2^/pig), slaughter weight (SW; 120 kg and 140 kg) and their interaction on average temperature (T) and relative humidity (RH) inside the compartments during winter.

Variable	Treatment							
	0.44 m^2^/pig		0.50 m^2^/pig			*p*-Value		
	120 kg	140 kg	120 kg	140 kg	SEM	SA	SW	SA × SW
Loading								
T, °C	4.02	4.9	3.5	3.6	1.7	0.17	0.35	0.21
RH, %	84.5	78.7	83.2	81.6	3.8	0.59	0.60	0.82
Wait-at-farm								
T, °C	6.2	7.2	5.6	6.1	3.0	0.39	0.47	0.34
RH, %	82.5	82.4	84.8	82.2	3.9	0.43	0.49	0.12
Transport								
T, °C	6.2	6.5	6.5	5.6	1.5	0.72	0.78	0.96
RH, %	80.9	82.9	85.5	82.7	3.5	0.34	0.46	0.36
Wait-at-abattoir								
T, °C	11.3	6.8	9.9	7.8	1.9	0.21	0.88	0.42
RH, %	73.7	87.0	79.7	85.8	3.9	0.72	0.71	0.53

**Table 10:** Effects of space allowance (SA; 0.44 m^2^/pig and 0.50 m^2^/pig), slaughter weight (SW; 120 kg and 140 kg) and their interaction on drinking and fighting behaviours during lairage in winter.

Variable	Treatment							
	0.44 m^2^/pig		0.50 m^2^/pig			*p*-Value		
	120 kg	140 kg	120 kg	140 kg	SEM	SA	SW	SA × SW
Drinking								
Latency to drink ^a^, s	39 [9–175]	72 [17–332]	93 [21–411]	93 [21–411]		0.43	0.65	0.56
Time spent drinking, %	22.7	13.4	19.0	17.5	4.0	0.96	0.15	0.29
Drinking bouts, n	78.5	48.8	69.3	60.5	14.0	0.92	0.13	0.39
Fighting								
Latency to fight, min	11.4	8.7	6.0	7.2	2.4	0.18	0.76	0.42
Time spent fighting, %	6.1	16.4	20.4	7.7	8.3	0.75	0.89	0.20
Total fights, n	13.3	14.3	28.5	13.5	6.0	0.25	0.27	0.21

^a^ Data were log-transformed, and values are reported as back-transformed least-square mean with the limits of the confidence interval in square brackets.

**Table 11:** Effects of space allowance (SA; 0.44 m^2^/pig and 0.50 m^2^/pig), slaughter weight (SW; 120 kg and 140 kg), and their interaction on average blood haematocrit, lactate, and creatine kinase (CK) concentrations at slaughter during winter.

Variable	Treatment							
	0.44 m^2^/pig		0.50 m^2^/pig			*p*-Value		
	120 kg	140 kg	120 kg	140 kg	SEM	SA	SW	SA × SW
Haematocrit, %	37.44	35.40	36.38	37.53	3.38	0.74	0.79	0.34
Lactate, mmol/L	11.18	12.64	11.58	11.89	0.89	0.83	0.29	0.48
CK, log UI/L	3.94	3.96	3.86	4.00	0.12	0.84	0.49	0.60

**Table 12:** Effects of space allowance (SA; 0.44 m^2^/pig and 0.50 m^2^/pig), slaughter weight (SW; 120 kg and 140 kg) and their interaction on carcass and pork quality traits as assessed in the *longissimus* muscle (LM) and *semimembranosus* (SM), and *adductor* (AD) muscles during winter.

Variable	Treatment							
	0.44 m^2^/pig		0.50 m^2^/pig			*p*-Value		
	120 kg	140 kg	120 kg	140 kg	SEM	SA	SW	SA × SW
HCW ^a^, kg	96.6	113.2	96.2	113.0	0.98	0.78	<0.001	0.94
Carcass yield, %	80.2	80.4	80.1	80.4	0.47	0.84	0.56	0.88
Lesion score ^b^	1.66	1.74	1.77	1.67	0.18	0.85	0.91	0.40
LM								
pHu	5.79	5.76	5.75	5.71	0.07	0.03	0.10	0.62
L*	50.90	51.81	50.77	51.60	0.51	0.74	0.11	0.94
a*	4.98	3.54	3.15	3.27	0.52	0.12	0.63	0.41
b*	11.23	11.35	10.76	11.10	0.43	0.20	0.40	0.69
Drip loss ^c^, %	2.55	2.52	2.74	2.87	0.27	0.28	0.85	0.72
SM muscle								
pHu	5.82	5.77	5.82	5.74	0.04	0.55	0.05	0.55
L*	47.75	48.32	47.44	48.69	0.39	0.94	0.04	0.40
a*	2.21	2.54	2.26	2.14	0.32	0.60	0.74	0.51
b*	8.77	9.14	9.00	9.14	0.19	0.56	0.21	0.57
EC ^d^, ms	5.71	5.67	5.83	5.79	0.23	0.49	0.81	0.97
AD muscle								
pHu	5.97	6.06	5.93	5.86	0.12	0.23	0.91	0.42

^a^ HCW: Hot carcass weight. ^b^ Based on photographic charts (from 1: none to 5: severe; [36]). ^c^ Percentage of drip loss was calculated by the equation: [% drip loss = −0.1 + (0.06 × mg fluid)] [10]. ^d^ EC: Electrical conductivity measured by the Pork Quality Meter.

## Data Availability

Not applicable.
