# Correction: Gonçalves Vero et al. Transport of Pigs of Two Market Weights at Two Space Allowances: Effects on Behaviour, Blood Parameters, and Meat Quality under Summer and Winter Conditions. *Animals* 2023, *13*, 2767

**DOI:** 10.3390/ani14050810

**Published:** 2024-03-06

**Authors:** Jessica Gonçalves Vero, Nicolas Devillers, Ana Maria Bridi, Kyle A. T. Moak, Gizella Aboagye, Guilherme Agostinis Ferreira, Jansller Luiz Genova, Sabine Conte, Luigi Faucitano

**Affiliations:** 1 Agriculture and Agri-Food Canada, Sherbrooke Research and Development Centre, 2000 College Street, Sherbrooke, QC J1M 0C8, Canada; jgveroo@gmail.com (J.G.V.); nicolas.devillers@agr.gc.ca (N.D.); sabine.conte@agr.gc.ca (S.C.); 2 Departamento de Zootecnia, Universidade Estadual de Londrina, Londrina 86051-990, Brazil; ambridi@uel.br (A.M.B.); guilherme.agostinis@uel.br (G.A.F.); 3 Department of Animal Science, University of Saskatchewan, Saskatoon, SK S7N 5B4, Canada; kyle.moak@usask.ca; 4 European Food Safety Authority (EFSA), Via Carlo Magno 1A, 43126 Parma, Italy; gizi.aboagye@gmail.com; 5 Departamento de Zootecnia, Universidade Federal de Viçosa, Viçosa 36570-900, Brazil; jansllerg@gmail.com

## Error in Figure

In the original publication [1], there was a mistake in Figures 1 and 2 as published. In Figure 1 (a representation of the truck design, including compartment dimensions, used for transport during summer), the value of the surface of compartment 2 of the truck section was incorrect, i.e., a surface value of 9.17 m^2^ was indicated, while the real surface area was 7.99 m^2^. In Figures 1 and 2 (a representation of the truck design, including compartment dimensions, used for transport during winter), the heights of the two truck decks were also incorrect, i.e., they were actually 1.03 m and 1.07 m for the bottom and upper deck, respectively, instead of the quoted 0.60 m for each deck. These corrections are thus required (1) to confirm the correct calculation and indication of the available floor space in compartment 2 (m^2^/number of animals; Figure 1) and (2) as the height indicated in the published paper was unrealistic, since, considering the height of pigs used in this study (approx. 80 cm), this deck height would not allow them to enter the truck. These corrections will not change the interpretation of the within-truck climate results and will not change our justification for not being able to record video footage of in-transit behaviour due to the low deck height (page 16). Instead, the new information will give even more support to our interpretation of the results, removing the bias of an excessively low deck height. The corrected Figure 1 and Figure 2 appear below. The authors state that the scientific conclusions are unaffected. This correction was approved by the Academic Editor. The original publication has also been updated.

## Figures and Tables

**Figure 1:**
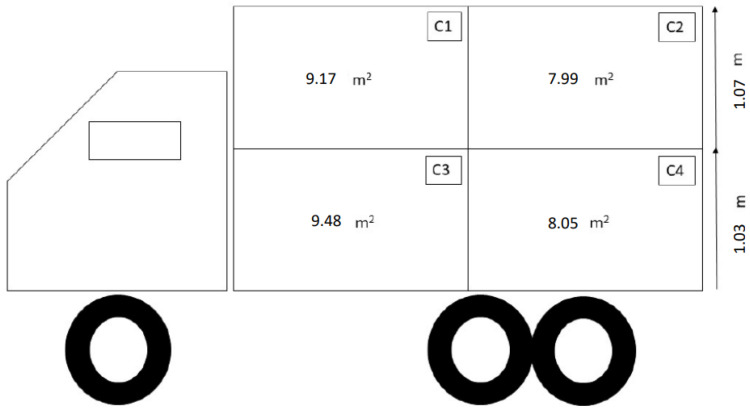
Representation of the truck design, including compartment dimensions, used for transport during summer.

**Figure 2:**
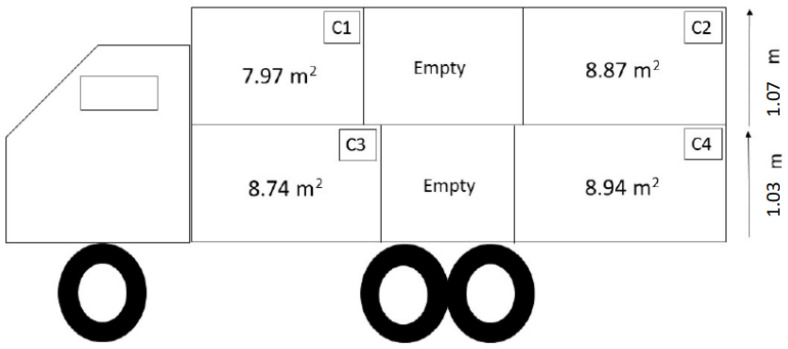
Representation of the truck design, including compartment dimensions, used for transport during winter.
